# Experimental investigation of oxygen diffusion in the peak and valley region of minibeam patterns during x‐ray irradiation

**DOI:** 10.1002/mp.17999

**Published:** 2025-08-08

**Authors:** Constantin Schorling, Evelyn Rauth, Christina Stengl, Joao Seco

**Affiliations:** ^1^ Department for Physics and Astronomy Heidelberg University Heidelberg Germany; ^2^ Division of Biomedical Physics in Radiation Oncology German Cancer Research Center (DKFZ) Heidelberg Germany; ^3^ Medical Faculty Heidelberg Heidelberg University Heidelberg Germany; ^4^ Division of Medical Physics in Radiation Oncology German Cancer Research Center (DKFZ) Heidelberg Germany; ^5^ Heidelberg Institute for Radiation Oncology (HIRO) National Center for Radiation Research in Oncology (NCRO) Heidelberg Germany; ^6^ Radiation Oncology Heidelberg University Hospital (UKHD) Heidelberg Germany

**Keywords:** minibeam irradiation, oxygen depletion measurements, spatial fractionation

## Abstract

**Background:**

Minibeam radiotherapy has demonstrated its potential to reduce normal tissue toxicity while maintaining tumor control. However, the underlying mechanisms behind this phenomenon remain unknown. Recent theoretical studies suggest a dose surrogate by diffusion of $H_{2}O_{2}$ into the valley regions.

**Purpose:**

The aim of this study is to experimentally investigate oxygen depletion and diffusion upon minibeam (MB) irradiation.

**Methods:**

A 3D‐printed water phantom with four sensors was developed to enable the real‐time, simultaneous measurement of oxygen concentration in the peak and valley. Water with 0%–11% $O_{2}$ and 0.1%/5.0% ${\mathrm{CO}}_{2}$ was irradiated with broad beam (BB) and MB characterized by peak and valley widths of 2 mm × 2 mm and 0.5 mm × 2 mm. The depletion was further compared in other chemical environments.

**Results:**

The oxygen depletion rates per dose in hypoxic water in the valley regions were found to be 3–7 times higher compared to the peaks or BB. This observation was found to be independent of oxygen concentration above 2 %, indicating oxygen depletion saturation. For MB, diffusion between peaks and valleys was observed. After a certain period, an equilibrium between diffusion and dose rate differences was established. Glutathione and HEPES as a medium increased the depletion further and distinguished MB from BB.

**Conclusions:**

A novel way of simultaneously measuring oxygen in the peak and valley of the MB dose pattern was introduced. The observed oxygen depletion saturation and diffusion between the peaks and valleys suggest the importance of oxygen in spatially fractionated radiotherapy studies, which is even greater for 5 mM glutathione compared to water.

## 1 INTRODUCTION

In spatially fractionated radiotherapy (SFRT), the irradiation is purposely blocked, creating regions of high and low doses.^1^, ^2^, ^3^ Alternating high‐ and low‐dose regions, also called peaks and valleys, can be achieved by introducing a collimator in the beam path.^4^, ^5^ Distributions with peak widths in the order of 100μm up to a few mm are called minibeams (MB).^1^, ^2^, ^3^ Recently, minibeam radiation therapy (MBRT) demonstrated a widening of the therapeutic window.^1^, ^6^, ^7^, ^8^, ^9^, ^10^, ^11^ Animal studies revealed robust control of tumor growth in mouse models of melanoma^9^ and increased long‐term survival in glioma‐bearing rats.^6^, ^10^, ^12^ Similar results were seen in glioma cells.^11^ However, the rationale for tumor control in addition to normal tissue preservation with MBRT has not been sufficiently established.^7^, ^13^ One proposed mechanism is based on the production of reactive oxygen species (ROS) by water radiolysis, which disseminate from the peak into the valley regions by diffusion.^14^ The production of ROS relies on the presence of molecular oxygen,^15^ which is mainly expressed by two reactions:^16^, ^17^, ^18^, ^19^, ^20^, ^21^

(1) $$\begin{array}{c} e_{\mathrm{aq}}^{-}+O_{2}⟶O_{2}^{-};\;k=1.9\times 10^{10}\;M^{-1}s^{-1} \end{array}$$

(2) $$\begin{array}{c} H^{\cdot }+O_{2}⟶{\mathrm{HO}}_{2}^{\cdot };\;k=1.8\times 10^{10}\;M^{-1}s^{-1}, \end{array}$$

with k being the reaction rate constant. With 2.6 $\frac{\text{molecules}}{100\;\text{eV}}$ the solvate electron ($e_{\mathrm{aq}}^{-}$) has a higher primary *G*‐value than the hydrogen radical ($H^{\cdot }$) with 0.6 $\frac{\text{molecules}}{100\;\text{eV}}$. In combination with the higher reaction rate constant, $e_{aq}^{-}$ is the dominating oxygen scavenger.^17^, ^18^ The $e_{aq}^{-}$ can react further with the hydroxyl radical:^18^, ^19^, ^20^, ^21^

(3) $$\begin{array}{cc} \; & e_{\mathrm{aq}}^{-}+{\mathrm{OH}}^{\cdot }⟶{\mathrm{OH}}^{-};\;k=3.0\times 10^{10}\;M^{-1}s^{-1}. \end{array}$$

Apart from oxygen, carbon dioxide (${\mathrm{CO}}_{2}$) is also an important parameter in physiology.^22^ It is used by cells to regulate their pH, and plays a role in the respiratory system. Moreover, it has been shown that tumors in mice breathing a mixture of oxygen and ${\mathrm{CO}}_{2}$ were significantly higher perfused, increasing also the radiosensitivity.^23^ ${\mathrm{CO}}_{2}$ reacts with water to form carbonic acid ($H_{2}{\mathrm{CO}}_{3}$).^24^, ^25^, ^26^ However, this is only true for under 1% of the ${\mathrm{CO}}_{2}$, while the rest stays in hydrated or aqueous form ${\left({\mathrm{CO}}_{2}\right)}_{aq}$.^26^ The carbonic acid dissolves further into bicarbonate (${\mathrm{HCO}}_{3}^{-}$) and subsequently into the carbonate ion (${\mathrm{CO}}_{3}^{2-}$). In water saturated with 5% ${\mathrm{CO}}_{2}$, the pH drops to 6.4.^27^ Additionally, when irradiated, ${\mathrm{CO}}_{2}$ undergoes radiolysis by being split into CO and $O_{2}$.^27^, ^28^, ^29^

The by‐products of the water radiolysis will react with ${\mathrm{CO}}_{2}$, ${\mathrm{HCO}}_{3}^{-}$, and ${\mathrm{CO}}_{3}^{2-}$.^30^

Despite the fact that chemical reactions and mechanisms have been described as surrogates for dose, the role of oxygen or carbon dioxide depletion in MB has only been the subject of theoretical investigations.^14^, ^27^, ^31^, ^32^ These Monte Carlo radiochemical studies were conducted in pure water; therefore, this study also uses pure water and hypoxic conditions as a benchmark for future theoretical studies. As a step toward in vitro environments, oxygen depletion was also studied in reducing and buffering agents. The aim of this work is to investigate experimentally the real‐time oxygen depletion in the peaks and valleys of MB patterns for different $O_{2}$ (0%–11%, i.e., hypoxia to physioxia^33^, ^34^), ${\mathrm{CO}}_{2}$ (0.1%/5.0%) and MB collimators (peak × valley width: 2 mm × 2 mm, 0.5 mm × 2 mm; comparison with BB).

## 2 MATERIALS AND METHODS

### 2.1 Phantom design

**FIGURE 1:**
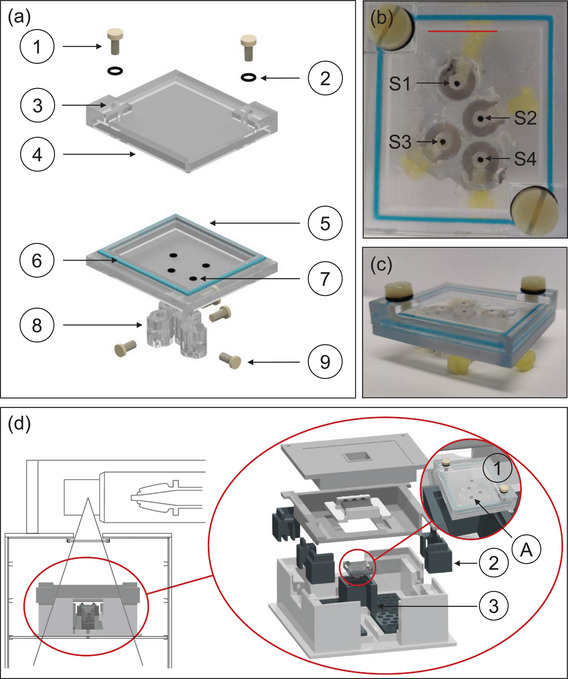
(a) Schematic of the phantom with oxygen sensors placed inside (7). M4 screws (1) and O‐rings (2) close holes (3) in the lid (4), which allow for filling the phantom. The blue ring (6) around the bottom part (5) is an additional sealing layer. Holders for the optical fibers (8) are attached at the bottom. Screws (9) secure the fibers in place. (b) Top view of the phantom with the front edge (red line) of the box. The sensors 1–4 are indicated by S1‐S4. (c) Side view of phantom after assembly. (d) The MB setup^2^ with the phantom (1) in the phantom holder (3) and additional adjusting components (3). During irradiation, S1 always faces the back of the setup.

The phantom was 3D printed with VeroClear (Stratasys Ltd., Israel), a PMMA‐like material, with inner dimensions of 30.9 mm (length) by 25.9 mm (width) by 3.91 mm (height). The transparency of VeroClear ensured optical reading with the sensors. The parts were printed on a J55 Prime 3D printer and an Objet500 Connex 1‐2‐3 3D printer (Stratasys, Israel). An additional sealing ring composed of Tango and VeroCyanV (Stratasys, Israel) with a Shore‐A degree of hardness of 85% was printed onto the lid to enhance the airtightness between lid and bottom part (see Figure 1a). The holders for the optical fibers were also 3D printed with VeroClear.

### 2.2 Oxygen sensors

Oxygen concentrations were measured with sensors cut from a TRACE Oxygen Sensor Foil (PyroScience GmbH, Germany) using a Cricut Joy (Cricut, USA). The mean diameter of the sensors was (1.06 ± 0.04) mm. For readout, the optical fibers were connected to the 4‐channel oxygen meter FireStingO2 (FSO2‐4, PyroScience GmbH). The sensors have been demonstrated to achieve a time resolution of approximately 400 ms. This allow us to capture oxygen depletion in the vicinity of the sensor during irradiation, as demonstrated in Jansen et al. (2021)^17^ for both conventional irradiation and ultra‐high dose rates.

### 2.3 Sensor placement for MB irradiation

For creating the MB dose pattern, the versatile collimator from Stengl et al. (2023)^2^ was used. The collimator consists of 1 mm wide tungsten plates alternating with 0.5 or 1.0 mm wide plastic plates to create valley and peak doses. The study investigates two different collimator configurations, with peak widths and valley widths of 2.0 mm × 2.0 mm and 0.5 mm ‐× 2.0 mm (hereafter referred to as 2x2 and 0.5x2, respectively). The placement of the sensors in the phantom was preceded by preliminary EBT‐XD Gafchromic film measurements (Ashland, USA). Three independent measurements were performed with each collimator configuration. An in‐house written script (MATLAB 2021b, MathWorks, USA), similar to Stengl et al. (2023),^2^ analyzed the films and calculated the mean full width at half maximum (FWHM) and center‐to‐center distance (CTC). Consequently, the sensors were positioned to allow measurement of both collimator configurations with a single phantom, with two sensors associated with the peak and valley regions respectively. The sensor positions were verified (see Section 2 Figure S1), which revealed a good alignment for all sensors except sensor S3 in the 2x2 pattern, which was therefore excluded. The sensor positions (1–4) were determined to be 9.28, 15.37, 19.44, and 22.40 mm to the front edge of the phantom (see Figure 1b,c). The uncertainty was approximated to be below 0.15 mm.

### 2.4 Irradiation setup

The irradiation was performed at a Faxitron MultiRad 225 X‐ray irradiation system (Precision X‐Ray, USA). A phantom holder was introduced to the setup from Stengl et al. (2023)^2^ as displayed in Figure 1d. The collimator was placed at a source‐to‐collimator distance of 21.8 cm, while the source‐to‐phantom distance was 23.9 cm. In order to ensure the correct position of the sensors in the peak and valley, a film was placed under the phantom during each MB irradiation. The phantom was irradiated with 200 kV tube voltage and 17.8 mA current. The dosimetry for the setup was performed similar to Stengl et al. (2023)^2^ (see Section S1). Furthermore, sensor‐specific dose rates were obtained. The phantom mean dose rate was obtained by integrating the dose over the whole phantom size and dividing the dose integral by the size. Comprehensive information concerning the sensor placement and sensor‐specific dose rates can be found in the Section S2.

### 2.5 Measuring oxygen depletion during irradiation

Double deionized water (Barnstead GenPure, Thermo Scientific, Germany) was placed in a Sci‐Tive hypoxic chamber (Baker Ruskinn, Ruskinn Technologies Ltd., UK), where nitrogen serves as air substitute, for at least two days to achieve (1.0–11.0)% $O_{2}$ and (0.1/5.0)% ${\mathrm{CO}}_{2}$. To mimic a radical scavenging and reducing intracellular environment, the oxygen depletion in different chemicals were investigated: Glutathione (GSH) (Sigma–Aldrich, Germany) was chosen to be investigated, as it is one of the primary intracellular scavengers and reducing agents.^35^ The concentration was set to be 5 mM in accordance to usual appearance in the human body.^36^ It was further rectified to a pH level of approx. 7. Additionally, the oxygen depletion was measured in HEPES (2‐[4‐(2‐hydroxyethyl)piperazine‐1‐yl]ethanesulfonic acid, 10 mM) (Sigma–Aldrich, Germany) to represent buffering agents. Both reagents were brought into the hypoxic chamber at 1% $O_{2}$ and 5% ${\mathrm{CO}}_{2}$. An overview of the $O_{2}$ and ${\mathrm{CO}}_{2}$ settings for the different measurements can be found in Table 1.

**TABLE 1:** Summary of the measurement settings.

Figure	Chemical	$O_{2}$ (%)	${\mathrm{CO}}_{2}$ (%)	Collimator
Figure 2	$H_{2}O$	1–14	0.1	—
Figure 3	$H_{2}O$	1	0.1	BB, 2x2, 0.5x2
Figure 4	$H_{2}O$, HEPES, GSH	1	5.0	BB, 2x2, 0.5x2
Figure 5a	$H_{2}O$	0–10	0.1, 5.0	BB
Figure 5b	$H_{2}O$	0–5	0.1	BB, 2x2
Figure 5c	$H_{2}O$	0–5	0.1	BB, 0.5x2

*Note*: The conenctration of HEPES as medium was set to 10 mM and for GSH, it was 5 mM.

Abbreviations: BB, broad beam, GSH, Glutathione.

A two‐point calibration of the sensors and cables was performed in normoxic and hypoxic water prior to the measurements. The raw values were subsequently corrected for temperature and pressure. After filling the phantom, a rest period was introduced to ensure stable conditions and to establish a steady state prior to irradiation. The phantom was then irradiated with BB, 2x2 and 0.5x2 MB. The oxygen depletion was measured during irradiation at a sampling rate of approximately one measurement per second. All measurements performed at approximately 1% $O_{2}$ were repeated three times, whereas the oxygen measurements for different initial oxygen concentrations were performed only once. Under hypoxic conditions, the phantom was irradiated for 20 min, while for varying oxygen concentrations, only 5 min BB and 2x2 MB intervals and 15 min intervals for 0.5x2 were assigned to an oxygen start value. The oxygen depletion gradients were obtained by fitting a linear function to the mean curves, subsequent to the attainment of equilibrium. The gradients were corrected for background variations that were recorded 120 s prior to each irradiation.

## 3 RESULTS

### 3.1 Dose rates at the sensor locations

The BB dose rate was established to be (6.12 ± 0.04) Gy/min. Furthermore, the dose rates for each sensor position and collimator pattern were determined (see Table 2). The (sensor‐associated) peak‐to‐valley dose ratio (PVDR) for 2x2 is approximately five, while for the 0.5x2 collimator it is close to 10. In addition, the average phantom dose rate for 0.5x2 is less than 0.5 Gy/min, while for 2x2 it is three times higher at approximately 1.5 Gy/min.

**TABLE 2:** Summary of the sensor dose rates for the different collimator configurations.

Collimator configuration	Sensor	Dose rate (Gy/min)
BB	S1–S4	6.120 ± 0.040
2.0 mm × 2.0 mm	S1 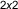 (Peak)	3.158 ± 0.032
	S2 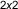 (Valley)	0.571 ± 0.041
	S4 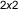 (Peak)	2.838 ± 0.060
	Mean (whole phantom)	1.517 ± 0.015
0.5 mm × 2.0 mm	S1 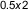 (Valley)	0.110 ± 0.011
	S2 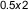 (Valley)	0.128 ± 0.012
	S3 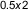 (Peak)	1.507 ± 0.022
	S4 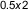 (Peak)	1.281 ± 0.035
	Mean (whole phantom)	0.457 ± 0.008

Doses were measured with EBT‐XD films and plotted against irradiation time to obtain the dose rates. S3 in 2x2 was excluded because it was not perfectly allocated in either a peak or a valley.

Abbreviations: BB, broad beam.

### 3.2 Oxygen depletion with BB and MB irradiation

**FIGURE 2:**
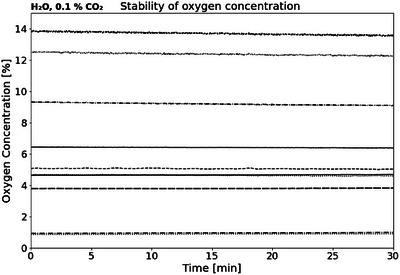
Stability measurements without irradiation of the phantom. For each background measurement (i.e., each line), the water was brought to the specific $O_{2}$ concentrations using the hypoxic chamber.

Stability measurements without irradiation showed no random diffusion, sensor drift or high background noise throughout the measurement period for all $O_{2}$ concentrations examined (Figure 2). Additionally, the stability was determined in water, HEPES and GSH at ${\mathrm{CO}}_{2}$ and showed a similar conditions (not shown).

First, the oxygen depletion was studied in dependence on the spatial beam structure in hypoxic water (1% $O_{2}$) devoid of carbon dioxide (0.1% ${\mathrm{CO}}_{2}$). For better comparison, all subsequent hypoxic oxygen depletion measurements were initially shifted to start at exactly 1% $O_{2}$. This is a reasonable simplification as all chemicals were initially brought to 1.

To compare the results obtained for water with different environments, the oxygen depletion per dose was measured and calculated in the same way as for the hypoxic water with 0% ${\mathrm{CO}}_{2}$ (Figure 4a,f). It is evident that the gradients observed are comparable to those of hypoxic water without ${\mathrm{CO}}_{2}$. For water with 5% ${\mathrm{CO}}_{2}$ the BB depletion was not significantly altered, in the 2x2 pattern only S1 (peak) showed a 6% slower depletion. For 0.5x2 the oxygen depletion of the peak sensors was increased, while for the valley sensors it was reduced, but this trend is only significant for S3 (peak).

**FIGURE 3:**
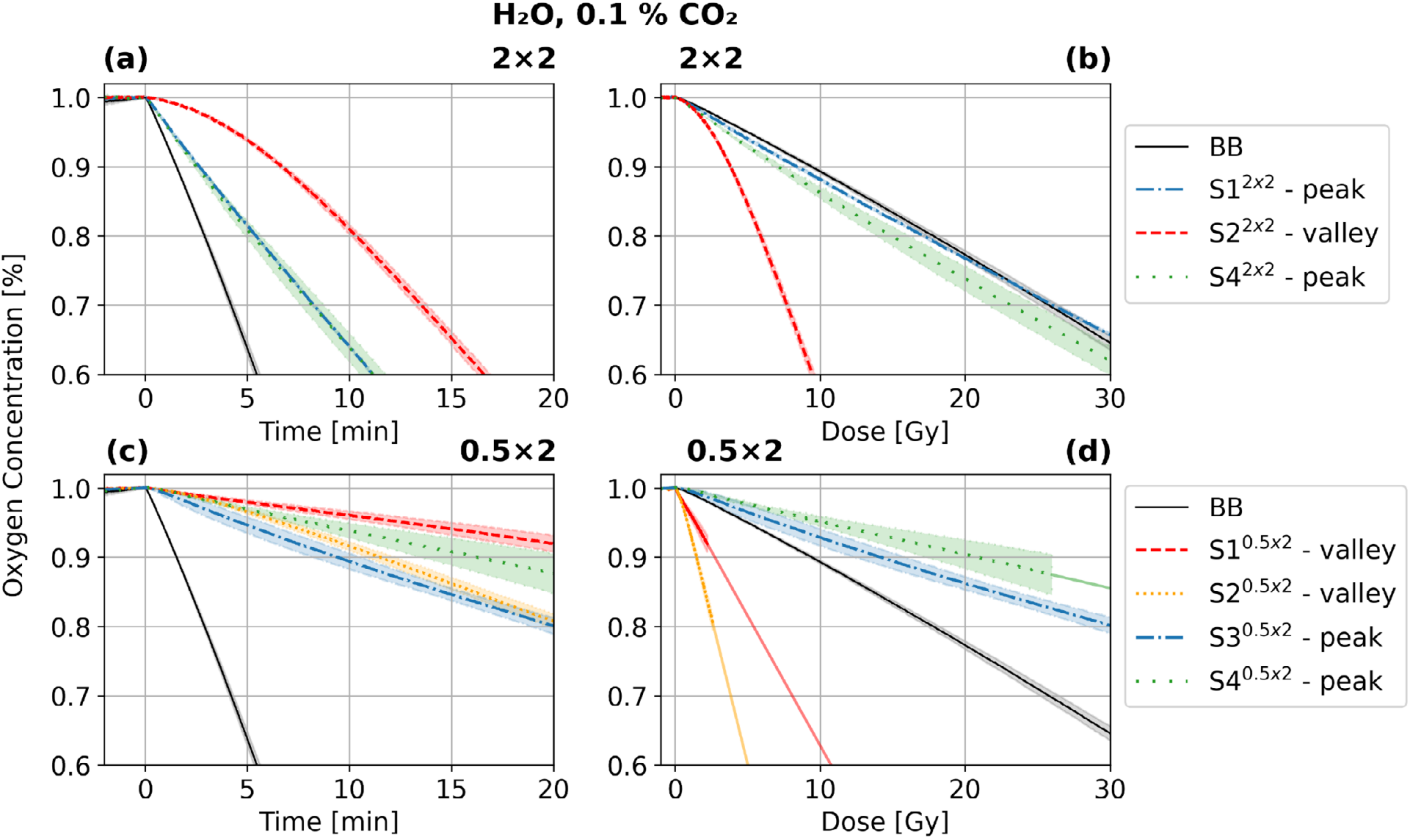
Mean oxygen depletion in hypoxic water (1% $O_{2}$, 0.1% ${\mathrm{CO}}_{2}$) for each sensor over time (a), (c), and dose (b), (d) for BB, 2x2 and 0.5x2 irradiation. The phantom was irradiated for 20 min. For S1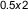, S2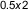 and S4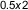, the dose‐dependent oxygen concentration was extrapolated by a linear line based on the depletion from the applied last 1 Gy irradiation. BB, broad beam.

**FIGURE 4:**
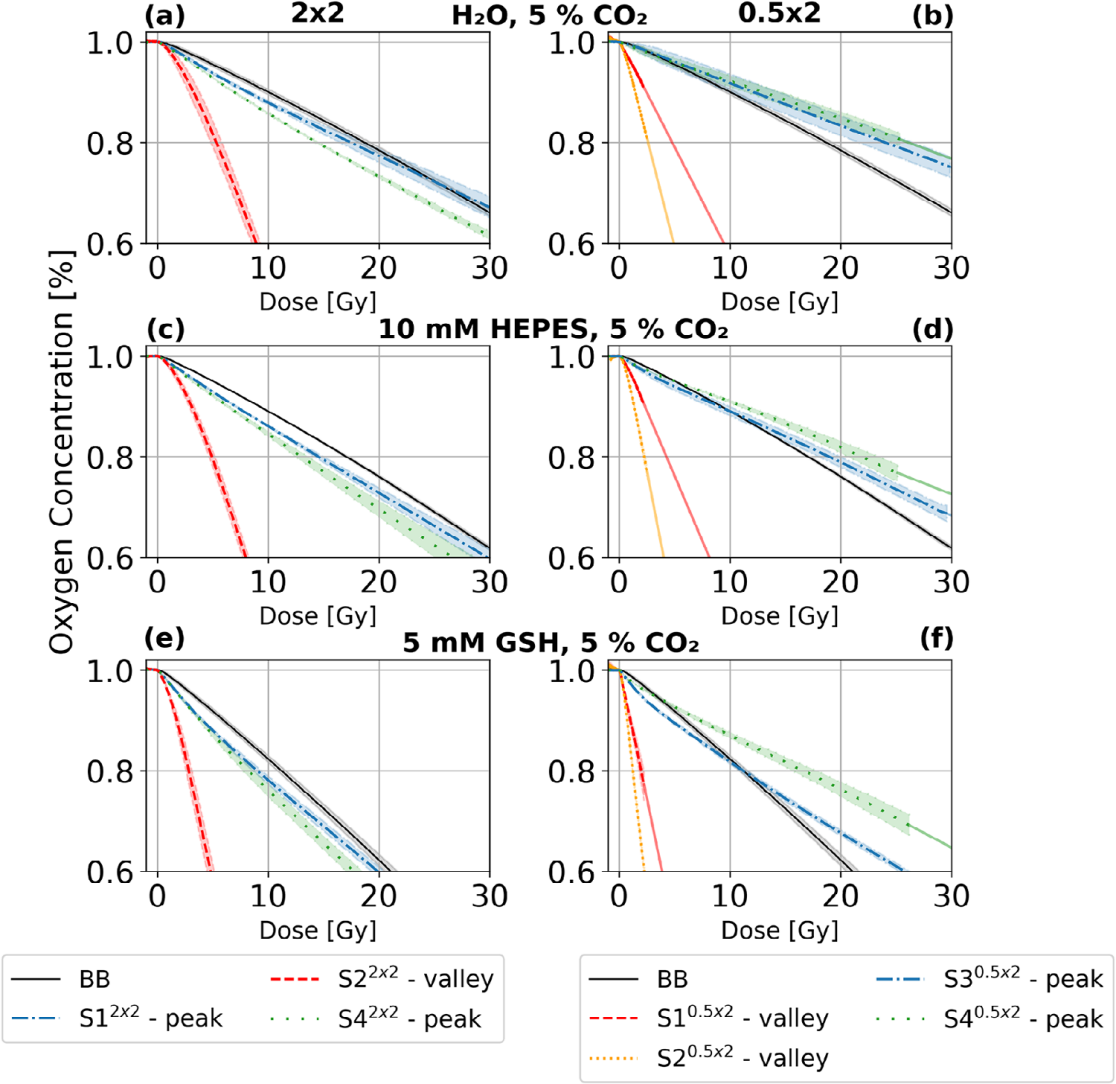
Mean oxygen concentration dependent on applied dose for different environments: $H_{2}O$ in (a) and (b); HEPES in (c) and (d); GSH in (e) and (f). All environments were set to 1.0% $O_{2}$ and 5.0% ${\mathrm{CO}}_{2}$. The phantom was irradiated for 20 min. For S1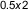, S2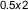, and S4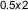 the dose‐dependent oxygen concentration was extrapolated by a linear line based on the depletion from the applied last 1 Gy irradiation. GSH, Glutathione.

For HEPES at 5% ${\mathrm{CO}}_{2}$, oxygen depletion was significantly increased for all sensors and irradiation structures. Although the relative deviation for the BB irradiation is still small at about 4%, it becomes more significant at 12%–57% for the MB irradiation. In general, a higher depletion amplification was observed for the peak sensors than for the valley sensors, so that the peak‐valley deviation gap was reduced. For GSH at 5% ${\mathrm{CO}}_{2}$, a significant increase in depletion was found, with at least 57% higher depletion rates. Here the valley sensors detected an even higher increased depletion than the peak sensors.

**FIGURE 5:**
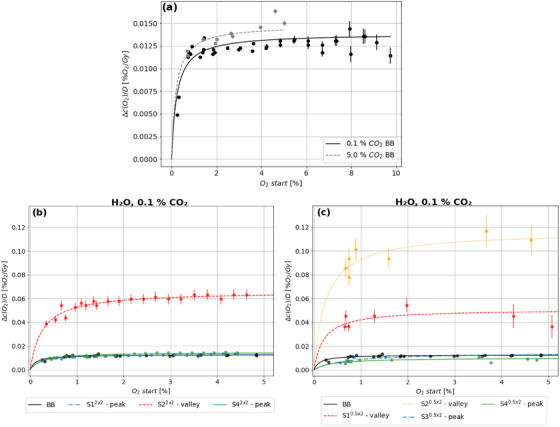
Michaelis–Menton fits of oxygen depletion per dose as a function of $O_{2}$ start. The Michaelis–Menton fits for water with 0.1% ${\mathrm{CO}}_{2}$ and 5.0% ${\mathrm{CO}}_{2}$ irradiated with BB were compared (a); in addition, for 0.1% ${\mathrm{CO}}_{2}$ BB was compared with 2x2 (b) and 0.5x2 (c) collimator patterns.

The oxygen depletion per dose can be plotted against varying oxygen concentrations and fitted with Michaelis–Menten kinetics (Figure 5a,c).^37^ In this context, the substrate concentration is associated with $c(O_{2})$, and $V_{max}$ with the maximum rate of oxygen depletion, resulting in $\frac{\Delta c(O_{2})}{D}=\frac{V_{max}\cdot c\left(O_{2}\right)}{k+c(O_{2})}$. For BB and oxygen concentrations above 2%, $c(O_{2})/D$ is found to be independent of the oxygen concentration, regardless the ${\mathrm{CO}}_{2}$ concentration. Furthermore, at 5% ${\mathrm{CO}}_{2}$, there is a 7% higher depletion for physioxic conditions. Similar to BB, both, peak and valley of the MB exhibit a plateau for $c(O_{2})/D$ above 2% $O_{2}$. In contrast, the valleys demonstrated a significantly higher oxygen depletion per dose. This phenomenon is particularly evident in the 0.5x2 valleys. $c(O_{2})/D$ of these valleys are 4–9 times higher than those of the peaks and the BB, for 2x2 it is almost five times higher.

## 4 DISCUSSION

This study focused on the simultaneous measurement of oxygen concentration in the peaks and valleys of a MB pattern during irradiation. Although the effect of MB irradiation on animals has already been subject to studies^5^, ^10^, ^38^ and there are several hypotheses, the underlying mechanism of the minibeam effect is still unknown.^1^, ^8^, ^14^, ^39^, ^40^ The minibeam effect has been shown for collimator configurations with the valley width being two to four times larger than the peak width.^2^, ^5^, ^7^, ^41^, ^42^ Therefore, the 0.5 mm × 2.0 mm and 2.0 mm × 2.0 mm collimator configurations were chosen. The positioning of the sensors in the peaks and valleys was successful, albeit with the exclusion of S3. This is corroborated by (sensor‐associated) PVDR of 5–14, which are consistent with the findings reported in the relevant literature.^2^, ^42^ The observed stability of the oxygen concentration in the absence of irradiation is consistent with the findings reported in the study by Jansen et al.,^17^ in which a phantom fabricated from the same material was used.

### 4.1 Oxygen depletion in hypoxic water

In the analysis of BB irradiation, a linear oxygen depletion gradient was observed from the onset. This observation aligns with the reported findings.^13^, ^43^, ^44^ These studies indicated a linear relationship between the duration of irradiation and oxygen depletion, irrespective of the specific irradiation type. Furthermore, the sensors exhibited a uniform response, which was anticipated given the homogeneous irradiation conditions. In the 2x2 configuration, there was a pronounced quadratic trend in the initial minutes for the peak sensors, and a particularly distinct inverse quadratic trend for the valley sensors. Similar observations can be seen for the 0.5x2 pattern. This phenomenon cannot be attributed solely to closed, noninteracting peak and valley systems, characterized by a constant dose rate difference, as these systems would also be expected to show a linear oxygen depletion, as shown for BB throughout the whole measurement period. Subsequently, after approximately 7 min, a linear trend emerged, suggesting that oxygen depletion gradients resulting from dose rate differences are compensated by diffusion. The 2x2 valley is characterized by about five times higher oxygen depletion per dose than the peaks, and the PVDR is also approximately fivefold. For 0.5x2, the valley gradients are 6 to 12 times higher than the peak ones, whereas the PVDR is 10 to 13.5 times higher. Conclusive deductions concerning the diffusion process necessitate a comprehensive understanding of dose‐rate‐specific oxygen depletion, a concept that was previously explored by Jansen et al.^17^ However, the study's primary focus was on ultra‐high dose rates, with the lowest investigated dose rate being approximately 0.1 Gy/s, which aligns with our BB irradiation. Nevertheless, the MB dose rates examined in our study range from 1/2 to 1/55 of this. Indeed, that the gradients of the 2x2 peaks are of the order of BB and the 0.5x2 peaks are lower is considered to be the result of dose rate dependencies as well as diffusion, which is dependent on PVDR and peak and valley width. The disparities observed among the peak sensors and between the valley sensors are associated with the position on the phantom (see Section S2, Figure S1). The oxygen concentration per dose for the valleys of 0.5x2 is extrapolated in Figures 3 and 4, as at this positions 0.4% $O_{2}$ were not depleted within 20 min, which demonstrates again the low dose rates in the valleys.

### 4.2 Oxygen depletion in other chemical environments

In the presence of ${\mathrm{CO}}_{2}$, the pH value of the water changes and it becomes acidic.^27^ An acidic environment favors $H^{\cdot }$, which is a scavenger for ${\mathrm{OH}}^{\cdot }$.^27^ According to Reaction 3, there will be more $e_{aq}^{-}$ present when there is less of the hydroxyl radical. Subsequently, the higher amount of available $e_{aq}^{-}$ leads to an increase in oxygen scavenging according to Reaction 1, and therefore a faster decrease in oxygen. Furthermore, ${\mathrm{CO}}_{2}$ and its by‐products from radiolysis and dissolving into carbonic acid can also scavenge ${\mathrm{OH}}^{\cdot }$ (see Section S3), which adds to the described chain reaction leading to a faster decrease in oxygen. However, one of the radiolysis products of ${\mathrm{CO}}_{2}$ is oxygen,^27^ increasing the amount of oxygen again and thus slowing the oxygen depletion down. In addition, ${\mathrm{CO}}_{2}$ and its by‐products from radiolysis and dissolution into carbonic acid can react with $e_{aq}^{-}$ ^18^, ^30^, ^45^ (see Section S3), which leaves less $e_{aq}^{-}$ for the reaction with oxygen according to Reaction 1. Therefore, the decrease in oxygen would be slowed down. We found nonsignificant changes in the oxygen depletion per dose of hypoxic water with 5% ${\mathrm{CO}}_{2}$ irradiated with BB, compared to water without ${\mathrm{CO}}_{2}$ (Figure 4a,b). It is noticeable that for 0.5x2 the peaks tend to deplete faster, while the valleys show the opposite behavior. The increased depletion in the peaks is thought to be caused by increased $e_{aq}^{-}$ availability and additional ${\mathrm{OH}}^{\cdot }$ scavenging. In contrast, in the valleys, oxygen depletion is reduced at 5% ${\mathrm{CO}}_{2}$ because ${\mathrm{CO}}_{2}$ and its by‐products react with $e_{aq}^{-}$, limiting its availability for oxygen reduction. However, it should be noted that no significant changes were found for 2x2, where peak and valley widths are suspected to be key contributors and need to be investigated further.

As a step toward in vitro studies, oxygen depletion was investigated in a radical scavenging and reducing intracellular environment (GSH) and a buffering agent (HEPES). Although the oxygen depletion for BB in HEPES was the same at 5% ${\mathrm{CO}}_{2}$ (Figure 4c,d) compared to water without ${\mathrm{CO}}_{2}$, this changed for MB. Here, the sensors detected an increase of between 12% and almost 60%, with the peaks tending to exceed the valleys. HEPES competes with oxygen for $e_{aq}^{-}$, but ${\mathrm{CO}}_{2}$ can also scavenge ${\mathrm{OH}}^{\cdot }$, leading to a complex interplay of reactions. As ROS production is generally less pronounced in the valley regions, and HEPES is less reactive to other radicals than $e_{aq}^{-}$, smaller increases may be seen while the peaks are more pronounced. As GSH is a reducing agent, it is expected to be oxidized to GSSG while reducing $H_{2}O_{2}$ and scavenging ${\mathrm{OH}}^{\cdot }$, $H^{\cdot }$, and $e_{aq}^{-}$.^46^, ^47^, ^48^ We observed an increase in oxygen depletion per dose for both BB and MB, as expected^48^(Figure 4e,f). Interestingly, while oxygen depletion was increased by approximately 60% for BB and most peak sensors, it was increased even further in all MB valleys, suggesting that the presence of GSH enhances oxygen depletion more efficiently in low‐dose regions, likely due to its interaction with radiolytic species such as $e_{aq}^{-}$ and ${\mathrm{OH}}^{\cdot }$. This could indicate a stronger radical‐driven oxygen depletion effect in valleys, where oxidative stress is more influenced by secondary reactions rather than direct ionization events.

### 4.3 Oxygen depletion in water at varying concentrations

Compared to hypoxic water, $\Delta c(O_{2})/Gy$ increases for oxygen concentrations up to 2% and reaches then a plateau for physioxic and normoxic conditions (Figure 5a,c). This is independent of the spatial beam structure and position within it. Jansen et al.^37^ found similar results for ultra high dose electron BB irradiation. The magnitude of the gradients for the BB can further be verified, as another study by Jansen et al.^17^ described similar gradients in this dose rate range. At a ${\mathrm{CO}}_{2}$ content of 5%, a 7% higher oxygen depletion per dose was found with BB for water with c($O_{2}$) above 2%. Under these conditions, this suggests a preference for the Reaction 1 over $O_{2}$ production by ${\mathrm{CO}}_{2}$ and $e_{aq}^{-}$ scavenging. The depletion per dose for the valleys follows that of BB and peaks at a high level. Again, no oxygen concentration dependence was found above 2%. The deviations of the two valleys in 0.5x2 correspond to the position of the sensors on the phantom: S1 is located furthest outwards and is surrounded by smaller peaks than S2 (see also Section S2.).

### 4.4 Limitations

A limitation of this study is the placement of the sensors directly in the peaks and valleys of the patterns. Although this was done successfully and clear differences were found, with smaller sensors and even more precise placement in the peaks and valleys, even greater differences between them can be expected. Although the behavior of buffering and reducing agents compared to water was examined, these environments do not fully capture the complexity of a cell and cell conglomerate. This is particularly true for oxygen supply and diffusion dynamics with biological, non‐irradiated adjacent environments. Furthermore, after MB irradiation, there would be an equalizing effect resulting from the depletion differences between peaks and valleys. However, investigating this in a closed system, as presented here, might not offer valuable insights toward in vitro and in vivo experiments, as in these environments, the radical removal process is predominant.^31^ This will require further research to confirm or disprove whether the radiochemical results obtained can be applied to biological systems.

## 5 CONCLUSION

This study measured oxygen depletion in the peaks and valleys of MB patterns during irradiation. Two collimator configurations with peak and valley widths of 2.0 mm × 2.0 mm and 0.5 mm × 2.0 mm were investigated. The scope of this study was to introduce experimental data on oxygen depletion and ROS production with MB irradiation. The presented data allows for future theoretical studies to have an experimental baseline in various environments, including hypoxia, physioxia, ${\mathrm{CO}}_{2}$, HEPES, and GSH. The oxygen depletion rates per dose in the valley regions were found to be significantly higher than in the peaks and with BB. This observation was predominantly independent of oxygen concentration and chemical environment. Furthermore, the occurrence of a quadratic dose response suggests a significant role for diffusion in the MB pattern as a whole, which subsequently reaches equilibrium with dose rate differences between the peaks and valleys. The novel findings on oxygen depletion saturation and diffusion between the peak and valley regions indicate an importance of $O_{2}$ for SFRT. The depletion observations in different chemical environments showed a change in radiolysis and diffusion behavior, making it imperative to bring the enhanced depletion in the valleys and diffusion observations into the biological context to elaborate on the mechanisms for subsequent cell damage from this found radio‐chemical contribution.

## 6 CREDIT AUTHORSHIP CONTRIBUTION STATEMENT

**Constantin Schorling**: Conceptualization, Methodology, Formal analysis, Investigation, Data curation, Visualization, Writing ‐ original draft, Writing ‐ review & editing **Evelyn Rauth**: Conceptualization, Methodology, Investigation, Writing ‐ original draft, Writing ‐ review & editing. **Christina Stengl**: Conceptualization, Methodology, Writing ‐ original draft, Writing ‐ review & editing, Supervision. **Joao Seco**: Conceptualization, Writing ‐ review & editing, Supervision.

## CONFLICTS OF INTEREST STATEMENT

The authors declare no conflicts of interest.

## Supporting information

Supporting Information
