# Supplementary material for: Experimental investigation of oxygen diffusion in the peak and valley region of minibeam patterns during x‐ray irradiation
Source: Med Phys. 2025 Aug 8;52(8):e17999. doi: 10.1002/mp.17999 (PMC12334874; doi:10.1002/mp.17999)
Supplement: Supplementary file 1 — Supporting Information [file MP-52-0-s001.pdf]

Supplementary Material

Experimental investigation of oxygen diffusion in the peak and valley region of minibeam patterns during X-Ray irradiation

Constantin Schorling, Evelyn Rauth, Christina Stengl, Joao Seco

.

Submitted to Medical Physics in April 2025

Supplementary Section S.I. Dosimetry

With RW3 plates, a Semiflex Ionization Chamber 0.125 cm^3 Type 31010 (PTW, Germany) was positioned at the water equivalent height of the films under the phantom. Performing four sets of five absolute charge measurements for 2 min, the broad beam dose rate was established to be (6.12 $\pm$ 0.04) Gy/min. By irradiating films with a homogeneous dose (broad beam) at the same position for (0, 0.5, 1, 2, 5, 7, 10, 12, 15, 20) min, the optical density of the films was mapped to the corresponding dose by a 4th order polynomial fit, yielding a calibration curve. Further, three sets of minibeam irradiation for (0, 1, 5, 10, 15, 20) min with each collimator configuration were measured. Using the dose rate and the calibration curve, the dose in the peak and in the valley was calculated.

Supplementary Section S.II. Minibeam Dose Pattern

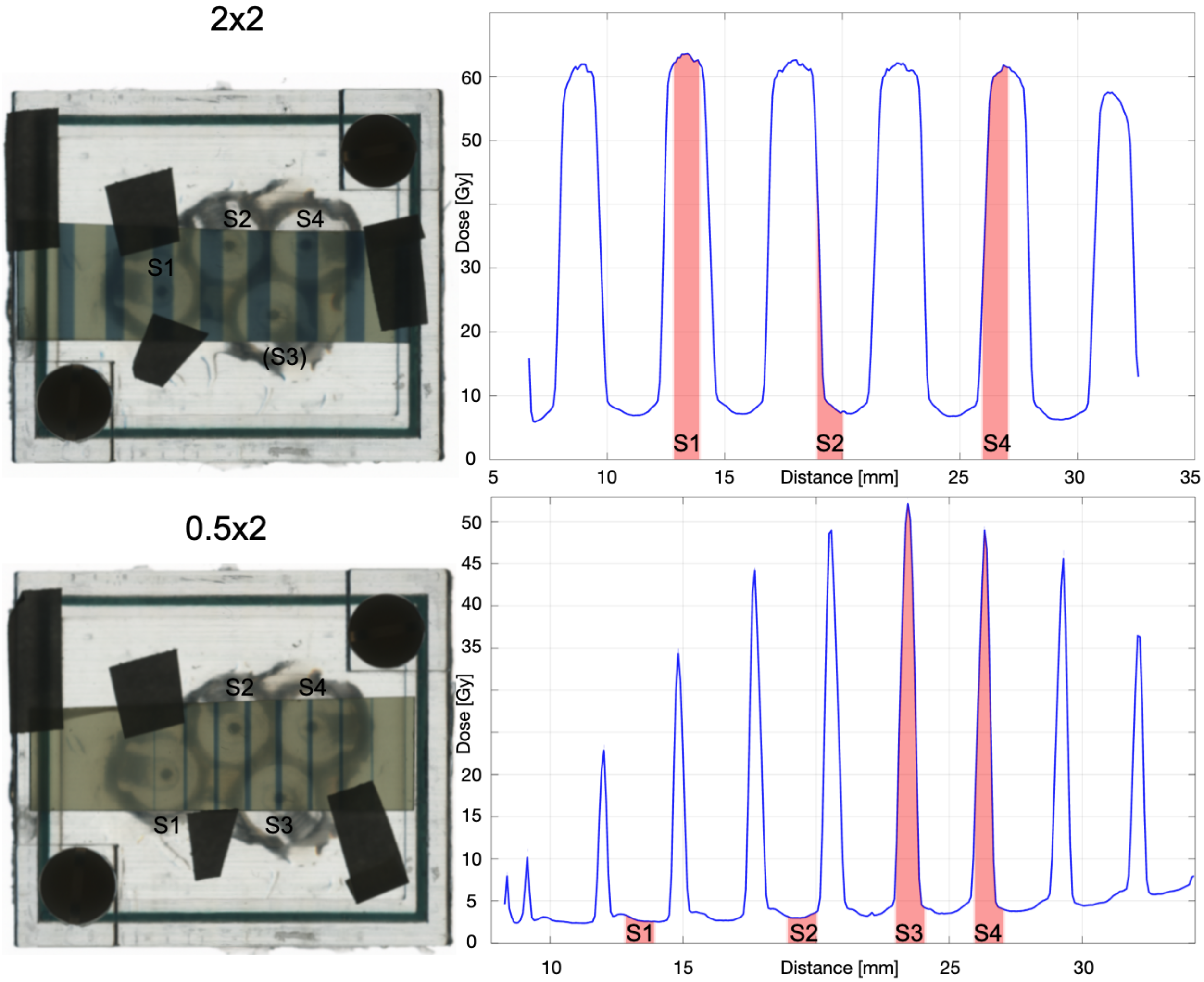

Supplementary Figure S1: Obtaining the minibeam dose pattern. Left: The scanned phantom with the labeled sensors and a film on top of the phantom to verfiy the sensor positions in the minibeam pattern. Right: Resulting dose pattern including the positioning of the sensors after film analysis. Top: Procedure for the 2.0 mm x 2.0 mm collimator configuration. Sensor S3 was excluded because it was not perfectly positioned in either a peak or a valley. Bottom: Procedure for the 0.5 mm x 2.0 mm collimator configuration.

Supplementary Section S.III. CO_2 reactions

When a carbonated solution, i.e. water containing carbon dioxide or carbonic acid, is irradiated, carbon dioxide will also undergo radiolysis by being split into CO and O_2[1,2]. Furthermore, the by-products of the water radiolysis (e.g. e^-_{aq}, $HO^\bullet$) will react with CO_2 and its by-products of dissolving carbonic acid[3]. Among others, the following reactions including their reaction rate constants k are possible[3–5]:

$$HO^\bullet + CO_3^{2-} \longrightarrow CO_3^{\bullet-} + OH^-; \qquad k = 3.9 \times 10^8\ M^{-1}s^{-1} \tag{1}$$

$$HO^\bullet + HCO_3^- \longrightarrow CO_3^{\bullet-} + H_2O; \qquad k = 8.5 \times 10^6\ M^{-1}s^{-1} \tag{2}$$

$$HO^\bullet + CO_2 \longrightarrow CO_3^{\bullet-}; \qquad k < 1.0 \times 10^6\ M^{-1}s^{-1} \tag{3}$$

$$HO^\bullet + CO \longrightarrow {}^\bullet CO_2H; \qquad k = (1.2 - 2.0) \times 10^9\ M^{-1}s^{-1} \tag{4}$$

$$e^-_{aq} + CO_2 \longrightarrow CO_2^{\bullet-}; \qquad k = 7.7 \times 10^9\ M^{-1}s^{-1} \tag{5}$$

$$e^-_{aq} + CO_3^{2-} \longrightarrow CO_2^{\bullet-} + 2\,OH^-; \qquad k = 3.9 \times 10^5\ M^{-1}s^{-1} \tag{6}$$

$$e^-_{aq} + CO_2^{\bullet-} \longrightarrow HCOO^- + OH^-; \qquad k = 9.0 \times 10^8\ M^{-1}s^{-1}. \tag{7}$$

References

[1] R. Kummler, C. Leffert, K. Im, R. Piccirelli, L. Kevan, and C. Willis. A numerical model of carbon dioxide radiolysis. *The Journal of Physical Chemistry*, 81(25):2451–2463, 1977.

[2] M. M. Ramirez-Corredores, G. Gadikota, E. E. Huang, and A. M. Gaffney. Radiation-Induced Chemistry of Carbon Dioxide: A Pathway to Close the Carbon Loop for a Circular Economy. *Frontiers in Energy Research*, 8, 2020.

[3] J. Vandenborre, L. Truche, A. Costagliola, et al. Carboxylate anion generation in aqueous solution from carbonate radiolysis, a potential route for abiotic organic acid synthesis on Earth and beyond. *Earth and Planetary Science Letters*, 564:116892, 2021.

[4] Z. Cai, L. Xifeng, Y. Katsumura, and O. Urabe. Radiolysis of Bicarbonate and Carbonate Aqueous Solutions: Product Analysis and Simulation of Radiolytic Processes. *Nuclear Technology - NUCL TECHNOL*, 136:231–240, 11 2001.

[5] G. V. Buxton, C. L. Greenstock, W. P. Helman, and A. B. Ross. Critical Review of rate constants for reactions of hydrated electrons, hydrogen atoms and hydroxyl radicals (.OH/.O-) in Aqueous Solution. *Journal of Physical and Chemical Reference Data*, 17:513–886, 1988.

List of Figures

S1 SetUp 2
